# Increased Intake of Omega-3 Polyunsaturated Fatty Acids Is Associated with Reduced Odds of Low Hand Grip Strength in Korean Adults

**DOI:** 10.3390/nu15020321

**Published:** 2023-01-09

**Authors:** Yoonjin Shin, Eugene Chang

**Affiliations:** 1 Korea Institute for Pharmaceutical Policy Affairs, Seoul 06708, Republic of Korea; 2 Department of Food and Nutrition, Gangneung-Wonju National University, Gangneung 25457, Republic of Korea

**Keywords:** omega-3, PUFA, hand grip strength, muscle strength, diet

## Abstract

Nutritional status is thought to be one of the modifiable risk factors for muscle health. This study investigates the association between dietary omega-3 polyunsaturated fatty acids (PUFA) intake and hand grip strength (HGS) in Korean adults. The cross-sectional analysis was performed on 18,278 participants aged ≥19 years enrolled in the Korea National Health and Nutrition Examination Survey from 2016–2019. Omega-3 PUFA consumption was positively linked to the dietary intake of nuts, fish, and shellfish in Korean adults. After adjusting for potential confounders, the results showed that increased omega-3 PUFA intake was associated with a decreased risk of low HGS (odds ratio (OR) for upper quartile (Q4) compared to Q1, men: OR = 1.42 (95% CI: 1.17–1.72), women: OR = 1.61 (1.37–1.89)). This inverse association was reported in people who did no resistance exercise or had an insufficient protein intake. In contrast, this association was not evident in adults who did resistance exercise or had sufficient protein intake. Furthermore, participants with hypertension or type 2 diabetes showed stronger associations between dietary omega-3 PUFA intake and HGS compared with other subgroups. These results suggest that dietary omega-3 PUFA intake positively related with HGS in Korean adults.

## 1. Introduction

Hand grip strength (HGS) is a simple and reliable indicator of whole muscle strength and a useful biomarker of health status [1]. A recent systematic review reported that HGS had predictive validity for reductions in cognition, mobility, and mortality in the elderly [2]. In addition, several studies have shown that low HGS in adults, not just the elderly, is associated with an increased risk of various chronic diseases, such as metabolic syndrome, type 2 diabetes, and cardiovascular disease (CVD) [3,4,5]. Therefore, HGS is a key health biomarker that should be managed, regardless of age.

Serval factors, such as gender, race, and hormone deficiency, may contribute to HGS [6,7,8]. In addition, adequate protein intake and regular physical activity, particularly resistance exercise, are known to be major modifiable factors for maintaining proper muscle function [9]. Nevertheless, conclusive evidence that protein intake has a major effect on muscle function is lacking [10,11]. The observed effects were inconsistent, and trials tended to have small sample sizes and be short in duration. Moreover, exercise is difficult to sustain because health and environmental factors often act as barriers [12]. Only about 40% of Korean adults practice regular walking, and fewer than 24.7% of adults perform resistance exercise at least 2 days per week [13]. Therefore, new strategies to prevent muscle strength decline are needed.

Omega-3 fatty acids are polyunsaturated fatty acids (PUFA) with anti-inflammatory properties that present in three major forms, namely alpha-linolenic acid (ALA; 18:3*n*-3), eicosapentaenoic acid (EPA; 20:5*n*-3), and docosahexaenoic acid (DHA; 22:6*n*-3). ALA is an essential dietary fatty acid that humans cannot synthesize; however, there are rarely activated endogenous pathways for the conversion of ALA to EPA and DHA [14]. Consequently, the levels of ALA, EPA, and DHA in the human body are primarily determined by dietary intake. ALA is mainly found in plant oils, such as nut and seed oils, while EPA and DHA are found in fish and other seafood [15]. Adequate intake of omega-3 PUFA has been associated with improved lipid profile control [16] and reduced risk of CVD and ischemic stroke [17,18].

Several papers describe the relationship between dietary omega-3 PUFA intake and muscle strength. In a study that examined the correlation between diet and grip strength, the consumption of fatty fish, a primary source of omega-3 PUFA, was positively associated with HGS in older adults [19]. In contrast, a study investigating the association between biochemical nutrient status markers and muscle strength found no significant association between plasma PUFA, including omega-3, and knee extension strength in older adults [20]. A prior intervention study also reported that long-term supplementation of omega-3 PUFA in the elderly, according to a multidomain lifestyle intervention, did not significantly affect grip strength [21]. These studies focused only on the elderly and did not consider important factors such as physical activity and protein intake that affect the relationship between dietary omega-3 PUFA intake and HGS.

Therefore, we investigated whether omega-3 PUFA consumption was associated with the risk of low HGS in adults of all age groups using the data from the Korea National Health and Nutrition Examination Survey (KNHANES). Furthermore, we conducted a stratified analyses to determine whether participants’ exercise, protein intake, and comorbidities, such as hypertension, diabetes mellitus, and dyslipidemia modified this relationship.

## 2. Materials and Methods

### 2.1. Study Population

This study used the data from the KNHANES VII (2016–2018) and the first year of the KNHANES VIII (2019–2021) because information about the participants’ HGS was only available for 2019. The KNHANES is an ongoing cross-sectional survey conducted by the Korea Disease Control and Prevention Agency (KDCA), which is designed to use complex, multistage, stratified, and probability cluster sampling to obtain nationally representative estimates. The survey includes a health interview, physical examination, and nutrition assessment. Detailed information about the survey is available on the website [22] (http://knhanes.cdc.go.kr [accessed on 1 December 2022]). The KNHANES was approved by the Institutional Review Board (IRB) of KDCA (IRB No: 2018-01-03-P-A and 2018-01-03-C-A). The participants’ informed consent requirement was waived because anonymous and de-identified information was used. Furthermore, our research procedures were performed in accordance with the Helsinki Declaration of 1975, as revised in 2008.

We initially selected the data from 32,379 individuals who had participated in the KNHANES. The exclusion criteria were as follows: individuals aged under 19 years (n = 6384), pregnant or lactating individuals (n = 205), those who reported an implausibly low or high intake of energy (<500 or >4000 kcal/day) (n = 3762), and those with missing HGS data (n = 1863) or other covariables (n = 1887). Ultimately, the analyses were performed using the data of 18,278 adults (7812 male and 10,466 female).

### 2.2. Measurement of Hand Grip Strength

HGS was measured by a digital hand dynamometer (Digital Grip Dynamometer, TKK-5401, Takei Scientific Instruments Co. Ltd., Tokyo, Japan). Measurements were performed with the participants standing with their arms fully extended to the side without touching the body. Participants were asked to hold the dynamometer with as much force as possible and alternate three times with each hand for less than 3 s; a rest interval of at least 60 s was allowed between each trial. The HGS value used in the analysis was the highest of the six measured values [23]. According to the recommendation of the Asian Working Group for Sarcopenia [24], low HGS was defined as below the 20th percentile of grip strength in the study population (<33.5 kg in men and <19.7 kg in women).

### 2.3. Assessment of Dietary Intake

Dietary data were collected using a single 24-h recall method by a trained dietitian at the subject’s home approximately 1 week after completing the health interview and physical examination. Energy and nutrient intake were calculated based on the Food Composition Table published by the Korean Rural Development Administration [25,26,27]. Adequate protein intake was defined as higher than the recommended nutrient intake of the Dietary Reference Intakes Koreans [28]. In this study, the nutrient intake was analyzed as the amount of intake per 1000 kcal of calories to exclude the effect of the difference in calorie intake on the intake of each nutrient as much as possible.

### 2.4. General Characteristics, Anthropometric Measurements, and Biochemical Variables

General information on gender, age, education (<high school, ≥high school), monthly income (<50th, ≥50th), alcohol consumption (yes, no), current smoker (yes, no), aerobic exercise (yes, no), and resistance exercise (yes, no) was collected using an interview administered questionnaire. Physical examinations were performed by trained medical staff following standardized procedures. Height and weight were measured in units of 0.1 cm and 0.1 kg, respectively, and body mass index (BMI) was calculated as weight divided by height squared (kg/m^2^). Blood pressure was measured using a mercury sphygmomanometer (Baumanometer^®^ Wall Unit 33 (0850), W. A. Baum Co. Inc., Copiague, NY, USA). Readings were taken three times on the participant’s right arm in a sitting position. The final blood pressure value was calculated as an average of the second and third blood pressure readings. All blood samples were collected in the fasting state and analyzed within 24 h for total cholesterol, high-density lipoprotein (HDL) cholesterol, triglyceride, hemoglobin A1c (HbA1c), fasting glucose, and high-sensitivity C-reactive protein (hs-CRP).

### 2.5. Statistical Analysis

All statistical analyses were performed using SAS^®^ statistical software v9.4 (SAS Institute Inc., Cary, NC, USA) with the significance level set at *p* < 0.05. The participants were categorized into quartiles (Q) according to their omega-3 PUFA intake. For descriptive statistics, the continuous variables were expressed as mean ± standard error, and the categorical variables were expressed as percentages. The general linear model and Cochran–Mantel–Haenszel analysis were used to determine differences in means and distribution of general characteristics and to test for linear trends according to omega-3 PUFA intake. Multivariate logistic regression analysis was applied to obtain odd ratios (OR) and 95% confidence intervals (CI) for the risk of low HGS. Statistically significant variables in the univariate analysis or potentially important factors associated with dietary intake and HGS were considered potential confounders and adjusted in the analyses. Model 1 was adjusted for age (years), BMI (kg/m^2^), menarche (women only), education level (<high school, ≥high), and monthly income status (< 50th, ≥50th). Model 2 was adjusted for the variables in model 1, plus alcohol intake (yes, no), smoking (yes, no), and aerobic exercise (yes, no). Model 3 was adjusted for the variables in model 2, plus energy intake (kcal/day), hypertension (yes, no), diabetes (yes, no), and dyslipidemia (yes, no).

## 3. Results

The participants were classified according to the quartiles of dietary omega-3 PUFA intake (Table 1). Adults with higher intakes of omega-3 PUFA tended to be slightly younger, more likely to participate in aerobic and resistance exercise, earn more, and be more educated. Participants with higher omega-3 PUFA intake exhibited lower hs-CRP concentrations than those with lower intakes. Moreover, dietary omega-3 intake was inversely associated with the prevalence of type 2 diabetes and dyslipidemia both in men and women. Additionally, dietary omega-3 intake showed an inverse relationship with the prevalence of hypertension in men.

Table 2 shows the daily dietary intake of the participants classified by their omega-3 PUFA intake. Omega-3 PUFA intake was positively related to protein and fat intake while inversely related to carbohydrate intake. As omega-3 intake increased, consumption of nuts, fish, shellfish, and plant and animal oils increased.

The multivariate analysis of the association between omega-3 PUFA intake and low HGS is shown in Table 3. After adjusting for confounding variables, adults with higher omega-3 PUFA intakes had significantly reduced odds of low HGS than those with lower omega-3 intake (men: OR = 1.42 [95% CI: 1.17–1.72]; women: OR = 1.61 [95% CI: 1.37–1.89]).

Table 4 presents the results of the stratified analyses according to exercise, protein intake, and comorbidities. Interestingly, an association was seen between omega-3 PUFA intake and low HGS in people who did not perform resistance exercise or had insufficient protein intake. In contrast, this association was not seen in those who participated in resistance exercise or had sufficient protein intake. Individuals with hypertension and type 2 diabetes showed stronger associations between omega-3 PUFA intake and low HGS than the other subgroups.

## 4. Discussion

This study investigated the association between omega-3 PUFA intake and the risk of low HGS. Results showed that dietary omega-3 PUFA intake is inversely associated with the risk of low HGS in adults. This association was more pronounced in participants who did no resistance exercise or had insufficient protein intake. In contrast, this association was less apparent in participants who did resistance exercise or had sufficient protein intake. In addition, individuals with hypertension or type 2 diabetes had stronger associations between omega-3 PUFA intake and low HGS.

An observational study of older adults in the United Kingdom found that each additional portion of fatty fish consumed per week resulted in an increased HGS [19]. Fatty fish is a major source of omega-3, and the association between omega-3 PUFA and HGS may partially explain these findings. Another study investigated the association between plasma PUFA and HGS in Iceland’s older adults with a 5-year follow-up [20]. Total omega-3, DHA, and EPA intakes were positively associated with HGS in a crude baseline model; however, no association was found in the longitudinal analysis. In an intervention study, healthy older adults who consumed omega-3 PUFA (1.86 EPA + 1.5 DHA) daily for 6 months had a higher HGS than those who did not [29]. However, daily supplementation with 1.3 g of PUFA for 3 months was not associated with improved HGS in the elderly [30]. Therefore, the current evidence regarding the relationship between omega-3 an HGS is inconsistent. In this study, we showed that dietary PUFA intake was significantly associated with a reduced risk of low HGS in Korean adults.

The European standard for omega-3 PUFA intake suggests 250–500 mg/day of EPA + DHA to prevent CVD [31]. The American Heart Association recommends consuming seafood once to twice a week to prevent CVD [18]; this is equivalent to 250 mg/day in terms of EPA + DHA [32]. According to KNHANES from 2013 to 2017, the average intake of EPA + DHA in Korean adults ranged from 155 to 503 mg/day, which is a higher intake compared to Western countries [33]. The recommended intake of omega-3 for maintaining or improving muscle health has not yet been proposed. However, a review article reported that supplementation of at least 2 g/day of omega-3 PUFA was associated with increased muscle function in healthy older adults, whether or not they exercised [34]. The article’s finding is consistent with this study’s results, which showed that participants in the upper Q (Q4) had a reduced risk of low HGS and consumed 2 g or more of omega-3 (men: >2.6 g, women: >2.1 g).

**Figure 1:**
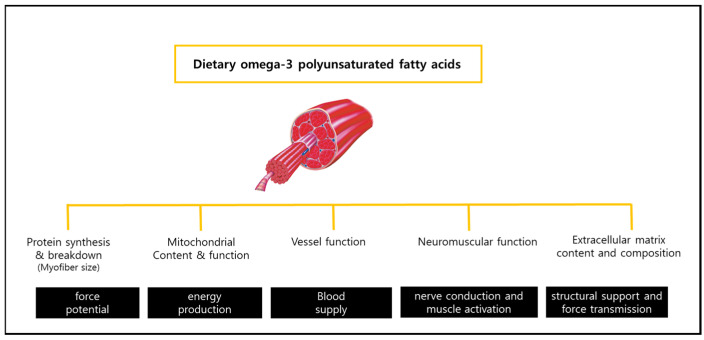
Potential effects of dietary omega-3 polyunsaturated fatty acids on muscle function.

The mechanisms mediating the beneficial effects of omega-3 PUFA on muscle strength have not been fully elucidated. However, it is speculated that omega-3 PUFA may increase the muscle synthesis and decrease the muscle proteolysis. Smith et al. found that 8 weeks of omega-3 PUFA supplement (4 g/day) increased the acute amino acid-induced activation of the mTOR-p70s6k signaling pathway and muscle protein synthesis in healthy older adults [35]. A review study found that fish oil-derived omega-3 PUFA attenuated muscle protein breakdown [36]. The positive effects of omega-3 PUFA may also be related to changes in myocytes themselves (mitochondrial content and function) and external factors (extracellular matrix content and composition, neuromuscular function) (Figure 1). Previous studies [37,38] demonstrated that dietary omega-3 PUFA promotes muscle mitochondrial biogenesis and content, and decreases total muscle and intramuscular triglyceride content. Walser et al. reported that 6 weeks of dietary supplementation with DHA (2.0 g/day) and EPA (3.0 g/day) induced brachial artery dilatation and increased blood flow in healthy middle-aged people [39]. Studies conducted in mice and rats have shown that fish oil improved motor and sensory nerve conduction speed and prevented the development of diabetic peripheral neuropathy [40,41]. Although these evidences have never been comprehensively evaluated in humans, it suggests that omega-3 PUFA may be an effective interventional strategy to reduce muscle strength loss.

A loss of muscle function can occur even with short-term reduced use. A previous study reported marked muscle loss after 4 days of immobilization, regardless of age [42]. It is difficult to recover muscle loss even with intensive physical rehabilitation [43], so it is recommended to do steady resistance-type exercises to maintain muscle strength. In addition, high protein intake is recommended; inadequate protein intake can lead to downregulation of muscle stem cell proliferation and upregulation of inflammatory responses [44]. In this study, the association between omega-3 PUFA intake and low HGS was found in people who did not perform resistance exercise or had an insufficient protein intake; in contrast, it was not seen in participants who did resistance training or had a sufficient protein intake. Therefore, increased omega-3 intake may be an alternative therapy for muscle health for people who do not participate in resistance training or have insufficient protein intake.

Furthermore, HGS is an indicator of frailty [45], which is associated with fatigue, reduced muscle mass, and high susceptibility to chronic diseases, such as type 2 diabetes and CVD. In healthy people, muscle strength reduces by ~10% every decade; loss of muscle strength starts at approximately 40–50 years of age, and periods of acute and chronic diseases accelerate the process [46,47]. This study showed that the association between omega-3 PUFA intake and HGS was stronger in individuals with hypertension, type 2 diabetes, and dyslipidemia than in those without these conditions. However, this relationship was not found in men with dyslipidemia, although the study’s Q1 subject group had the highest risk of low HGS. Therefore, high omega-3 PUFA intake might help reduce the risk of low HGS, even in individuals with hypertension, type 2 diabetes, and dyslipidemia.

To the best of our knowledge, this is the first study to examine the association between omega-3 PUFA consumption and HGS in adult populations based on a nationally representative sample. However, the study has some limitations. First, a cause-effect could not be established since the relationship between omega-3 PUFA and HGS was analyzed in a cross-sectional study. Further investigation is needed to clarify the causal relationship. Second, our dietary data were examined using a single 24-h dietary recall, which may not be sufficient to estimate usual dietary intake. However, in the 2009 KNHANES, only slight changes were observed when single (24 h) dietary recall was compared with data obtained over 2–10 days [48].

## 5. Conclusions

This study used data from the KNHANES to demonstrate that dietary omega-3 PUFA intake was inversely associated with HGS in adults aged ≥19 years. In particular, participants who did not partake in resistance exercise, consumed insufficient protein, or had hypertension or type 2 diabetes showed a stronger association between dietary omega-3 PUFA intake and HGS than other subgroups. Although we believe that additional longitudinal follow-up studies are necessary, we expect that this study’s findings can be used as a guideline for diets designed to promote muscle health.

## Figures and Tables

**Table 1:** General characteristics of the participants according to dietary omega-3 polyunsaturated fatty acids intake.

	Men					Women				
	Q 1 (*n* = 1953) <0.86 g	Q 2 (*n* = 1953) 0.86–1.55 g	Q 3 (*n* = 1953) 1.55–2.6 g	Q 4 (*n* = 1953) >2.6 g	Trend *p*	Q 1 (*n* = 2617) <0.65 g	Q 2 (*n* = 2617) 0.65–1.20 g	Q 3 (*n* = 2617) 1.20–2.09 g	Q 4 (*n* = 2617) >2.09 g	Trend *p*
Age (years)	54.8 ± 0.41	51.8 ± 0.38	50.0 ± 0.37	49.9 ± 0.37	<0.001	54.9 ± 0.34	51.2 ± 0.32	49.9 ± 0.31	50.2 ± 0.29	<0.001
Body mass index (kg/m^2^)	24.3 ± 0.08	24.4 ± 0.07	24.6 ± 0.07	24.7 ± 0.07	0.006	23.8 ± 0.07	23.4 ± 0. 07	23.5 ± 0. 07	23.3 ± 0.07	0.096
Systolic blood pressure (mmHg)	122.7 ± 0.36	121.2 ± 0.33	120.8 ± 0.33	120.1 ± 0.33	0.006	120.2 ± 0.37	117.5 ± 0.35	116.0 ± 0.33	116.4 ± 0.34	0.002
Diastolic blood pressure (mmHg)	76.6 ± 0.24	77.8 ± 0.22	78.0 ± 0.23	77.6 ± 0.23	0.137	73.8 ± 0.19	74.1 ± 0.18	73.9 ± 0.18	74.1 ± 0.18	0.151
Glucose (mg/dL)	105.6 ± 0.63	104.3 ± 0.58	102.7 ± 0.50	103.1 ± 0.53	0.122	100.1 ± 0.43	98.5 ± 0.42	97.9 ± 0.39	98.1 ± 0.40	0.431
HbA1c (%)	5.8 ± 0.02	5.8 ± 0.02	5.7 ± 0.02	5.7 ± 0.02	0.106	5.8 ± 0.02	5.7 ± 0.01	5.7 ± 0.01	5.7 ± 0.01	0.318
Total cholesterol (mg/dL)	187.0 ± 0.88	189.9 ± 0.85	191.1 ± 0.87	191.5 ± 0.84	0.008	192.1 ± 0.75	194.3 ± 0.72	194.0 ± 0.72	195.9 ± 0.73	<0.001
HDL cholesterol (mg/dL)	46.8 ± 0.26	47.9 ± 0.26	47.2 ± 0.25	47.8 ± 0.25	0.232	53.1 ± 0.24	54.9 ± 0.25	55.3 ± 0.25	55.7 ± 0.26	<0.001
Triglycerides (mg/dL)	156.1 ± 2.78	157.6 ± 2.84	157.7 ± 2.91	152.3 ± 2.90	0.282	120.4 ± 1.54	114.8 ± 1.68	113.7 ± 1.73	110.6 ± 1.45	0.035
LDL cholesterol (mg/dL)	110.7 ± 0.79	112.7 ± 0.77	114.5 ± 0.78	115.2 ± 0.75	0.001	115.4 ± 0.68	117.1 ± 0.66	116.5 ± 0.63	118.4 ± 0.66	0.000
Atherogenic index	3.2 ± 0.03	3.2 ± 0.03	3.2 ± 0.03	3.2 ± 0.03	0.803	2.8 ± 0.02	2.7 ± 0.02	2.7 ± 0.02	2.7 ± 0.02	0.172
hs-CRP (mg/L)	1.52 ± 0.07	1.18 ± 0.05	1.30 ± 0.06	1.25 ± 0.06	0.030	1.17 ± 0.04	1.08 ± 0.04	1.07 ± 0.04	1.00 ± 0.04	0.042
Current drinker (%)	1500 (76.8)	1621 (83.0)	1669 (85.4)	1663 (85.2)	<0.001	1566 (59.9)	1749 (66.8)	1802 (68.9)	1762 (37.4)	0.507
Current smoker (%)	651 (33.3)	633 (32.4)	653 (33.4)	563 (28.8)	<0.001	129 (4.9)	134 (5.1)	133 (5.1)	108 (4.1)	0.024
Aerobic exercise (%)	793 (40.6)	908 (46.5)	955 (48.9)	990 (50.7)	<0.001	949 (36.3)	1108 (42.3)	1121 (42.8)	1131 (43.2)	0.012
Resistance exercise (%)	469 (24.0)	578 (29.6)	599 (30.7)	614 (31.4)	<0.001	284 (10.9)	399 (15.3)	400 (15.3)	438 (16.8)	<0.001
Education (≧high school, %)	1247 (63.9)	1483 (75.9)	1569 (80.3)	1602 (82.0)	<0.001	1423 (54.4)	1733 (66.2)	1897 (72.5)	1935 (74.0)	<0.001
Monthly income (≧50th, %)	920 (47.1)	1151 (58.9)	1235 (63.2)	1269 (64.3)	<0.001	1211 (46.3)	1463 (55.9)	1553 (59.3)	1629 (62.3)	<0.001
Hypertension (%)	790 (40.5)	710 (36.4)	661 (33.9)	650 (33.3)	0.205	972 (37.2)	778 (29.7)	676 (25.8)	660 (25.2)	0.002
Type 2 diabetes (%)	359 (18.4)	297 (15.2)	265 (13.6)	254 (13.0)	0.015	365 (14.0)	280 (10.7)	231 (8.8)	233 (8.9)	0.022
Dyslipidemia (%)	929 (47.6)	889 (45.5)	938 (48.0)	878 (45.0)	0.042	892 (34.1)	834 (31.9)	775 (29.6)	814 (31.1)	0.033

HbA1c, Hemoglobin A1c; HDL, High-density lipoprotein; LDL, Low-density lipoprotein; PUFA, Polyunsaturated fatty acid. Values were expressed as means (SE) or number (%). Trend *p* obtained in general linear model analysis and Cochran-Mantel-Haenszel analysis with adjustment for age.

**Table 2:** Daily nutritional intake of the participants according to dietary omega-3 polyunsaturated fatty acids intake.

	Men					Women				
The Amount of intake per 1000 kcal	Q 1 (*n* = 1953)	Q 2 (*n* = 1953)	Q 3 (*n* = 1953)	Q 4 (*n* = 1953)	Trend *p*	Q 1 (*n* = 2617)	Q 2 (*n* = 2617)	Q 3 (*n* = 2617)	Q 4 (*n* = 2617)	Trend *p*
Energy (kcal)	1688.6 ± 14.08	2105.1 ± 15.65	2403.2 ± 17.11	2751.8 ± 19.21	<0.001	1233.6 ± 8.64	1548.2 ± 10.26	1762.3 ± 10.89	2070.1 ± 14.00	<0.001
Carbohydrate (g)	166.2 ± 0.84	154.6 ± 0.76	147.9 ± 0.76	138.7 ± 0.72	<0.001	178.08 ± 0.59	164.10 ± 0.58	155.43 ± 0.59	148.45 ± 0.58	<0.001
Protein (g)	32.4 ± 0.26	35.1 ± 0.21	37.4 ± 0.23	39.0 ± 0.23	<0.001	31.71 ± 0.21	35.40 ± 0.19	37.51 ± 0.19	38.52 ± 0.20	<0.001
Fat (g)	15.8 ± 0.21	19.6 ± 0.20	22.0 ± 0.21	25.7 ± 0.23	<0.001	15.86 ± 0.19	20.46 ± 0.19	23.26 ± 0.19	26.16 ± 0.20	<0.001
Omega-3 PUFA (g)	0.52 ± 0.004	1.20 ± 0.01	2.02 ± 0.01	4.51 ± 0.07	<0.001	0.34 ± 0.003	0.65 ± 0.005	0.99 ± 0.01	2.01 ± 0.03	<0.001
Alpha-linoenic acid (mg)	264.9 ± 3.30	494.7 ± 4.98	698.4 ± 6.95	1288.2 ± 22.91	<0.001	279.7 ± 2.90	518.2 ± 4.49	768.7 ± 6.65	1549.3 ± 24.19	<0.001
Eicosapentaenoic acid (mg)	18.8 ± 0.64	37.6 ± 1.05	63.7 ± 1.84	135.8 ± 4.20	<0.001	18.3 ± 0.50	37.1 ± 0.95	63.1 ± 1.63	122.2 ± 3.33	<0.001
Docosahexaenoic acid (mg)	35.3 ± 1.14	73.5 ± 2.03	124.8 ± 3.46	289.5 ± 9.41	<0.001	34.0 ± 0.87	73.5 ± 1.76	123.3 ± 3.09	256.9 ± 7.45	<0.001
Omega-6 PUFA (g)	2.8 ± 0.03	4.2 ± 0.04	5.1 ± 0.05	6.5 ± 0.07	<0.001	2.85 ± 0.03	4.27 ± 0.04	5.35 ± 0.04	6.67 ± 0.07	<0.001
Nuts (g)	2.4 ± 0.38	2.5 ± 0.22	3.4 ± 0.30	4.2 ± 0.25	<0.001	3.2 ± 0.31	4.3 ± 0.36	4.6 ± 0.34	6.2 ± 0.34	<0.001
Beans (g)	10.7 ± 0.62	20.4 ± 0.86	24.4 ± 1.07	24.0 ± 1.05	<0.001	10.9 ± 0.60	21.2 ± 0.97	26.7 ± 0.98	28.7 ± 1.10	<0.001
Oils in plant foods (g)	1.3 ± 0.05	2.4 ± 0.06	3.3 ± 0.07	4.7 ± 0.10	<0.001	1.5 ± 0.05	2.5 ± 0.06	3.5 ± 0.07	4.7 ± 0.08	<0.001
Fish and shellfish (g)	37.7 ± 1.90	52.6 ± 1.83	62.4 ± 1.89	73.0 ± 1.90	<0.001	44.2 ± 1.91	52.1 ± 1.66	61.2 ± 1.79	73.7 ± 1.81	<0.001
Eggs (g)	10.5 ± 0.48	15.0 ± 0.51	15.3 ± 0.49	15.3 ± 0.48	<0.001	12.4 ± 0.49	17.6 ± 0.52	18.7 ± 0.52	17.3 ± 0.47	<0.001
Oils in animal foods (g)	0.1 ± 0.01	0.1 ± 0.01	0.1 ± 0.02	0.2 ± 0.02	<0.001	0.1 ± 0.01	0.1 ± 0.02	0.1 ± 0.01	0.2 ± 0.02	<0.001

PUFA, Polyunsaturated fatty acid. Values were expressed as means (SE). Nutrient and food intake was represented as grams per 1000 kcal of energy. Trend *p* obtained in general linear model analysis with adjustment for age, body mass index, menarche (women only), alcohol intake, smoking, education level, income status, exercise, total energy intake, hypertension, diabetes, and dyslipidemia.

**Table 3:** Odds ratios (95% CI) of the effect of dietary omega-3 polyunsaturated fatty acids intake on low muscle strength.

	Men					Women				
	Prevalence (%)	Unadjusted	Model 1	Model 2	Model 3	Prevalence (%)	Unadjusted	Model 1	Model 2	Model 3
Q1	578 (29.6)	2.44 (2.08–2.86)	1.68 (1.41–2.00)	1.65 (1.38–1.97)	1.42 (1.17–1.72)	729 (27.9)	2.16 (1.88–2.48)	1.62 (1.40–1.87)	1.60 (1.38–1.85)	1.61 (1.37–1.89)
Q2	383 (19.6)	1.42 (1.20–1.68)	1.21 (1.01–1.45)	1.21 (1.01–1.46)	1.11 (0.91–1.34)	565 (21.6)	1.54 (1.34–1.77)	1.41 (1.21–1.64)	1.43 (1.22–1.65)	1.42 (1.22–1.66)
Q3	324 (16.6)	1.16 (0.97–1.37)	1.13 (0.94–1.36)	1.15 (0.94–1.39)	1.09 (0.90–1.32)	432 (16.5)	1.11 (0.95–1.28)	1.09 (0.94–1.28)	1.10 (0.94–1.29)	1.11 (0.95–1.30)
Q4	287 (14.7)	1.00 (Ref.)	1.00 (Ref.)	1.00 (Ref.)	1.00 (Ref.)	397 (15.2)	1.00 (Ref.)	1.00 (Ref.)	1.00 (Ref.)	1.00 (Ref.)
Trend *p*	<0.001	<0.001	<0.001	<0.001	0.002	<0.001	<0.001	<0.001	<0.001	<0.001

CI, confidence interval; PUFA, Polyunsaturated fatty acid. Prevalence was conducted by Cochran–Mantel–Haenszel analysis with adjustment for age. Odd ratios and 95% CIs were conducted by general linear model analysis. Model 1: adjusted for age, body mass index, menarche (women only), education level, and income status; Model 2: age, body mass index, menarche (women only), education level, income status, alcohol intake, smoking, and exercise; Model 3: age, body mass index, menarche (women only), education level, income status, alcohol intake, smoking, exercise, total energy intake, hypertension, diabetes, and dyslipidemia.

**Table 4:** Odds ratios (95% CI) of dietary omega-3 polyunsaturated fatty acids intake on low muscle strength considering resistance exercise, protein intake, and comorbidities.

	Men					Women				
	Q 1 (*n* = 1953)	Q 2 (*n* = 1953)	Q 3 (*n* = 1953)	Q 4 (*n* = 1953)	Trend *p*	Q 1 (*n* = 2617)	Q 2 (*n* = 2617)	Q 3 (*n* = 2617)	Q 4 (*n* = 2617)	Trend *p*
Aerobic exercise										
Yes	1.45 (1.07–1.96)	1.46 (1.09–1.95)	0.15 (0.86–1.55)	1.00 (ref.)	0.017	1.39 (1.07-1.81)	1.29 (1.00–1.65)	1.04 (0.80–1.34)	1.00 (ref.)	0.001
No	1.87 (1.43–2.46)	1.23 (0.93–1.62)	1.43 (1.08–1.89)	1.36 (1.03–1.80)	0.050	1.83 (1.46–2.30)	1.59 (1.27–2.00)	1.22 (0.97–1.53)	1.06 (0.84–1.33)	<0.0001
Resistance exercise										
Yes	1.30 (0.89–1.90)	1.07 (0.74–1.56)	1.00 (0.69–1.47)	1.00 (ref.)	0.169	1.12 (0.70–1.80)	1.37 (0.90–2.09)	1.09 (0.70–1.69)	1.00 (ref.)	0.565
No	2.35 (1.72–3.20)	1.84 (1.35–2.50)	1.87 (1.37–2.55)	1.68 (1.23–2.30)	0.017	2.26 (1.63–3.13)	1.96 (1.42–2.70)	1.52 (1.10–2.11)	1.39 (1.00–1.92)	<0.0001
Adequate protein intake										
Yes	1.32 (0.81–2.17)	1.17 (0.75–1.81)	1.33 (0.88–2.01)	1.00 (ref.)	0.268	0.94 (0.56–1.58)	0.87 (0.59–1.29)	0.96 (0.67–1.37)	1.00 (ref.)	0.311
No	2.32 (1.66–3.25)	1.83 (1.31–2.55)	1.78 (1.27–2.49)	1.71 (1.22–2.40)	0.014	1.89 (1.43–2.50)	1.74 (1.32–2.30)	1.31 (0.99–1.74)	1.13 (0.85–1.49)	<0.0001
Hypertension										
No	1.32 (1.03–1.70)	1.04 (0.81–1.33)	1.00 (0.78–1.28)	1.00 (ref.)	0.041	1.38 (1.13–1.69)	1.31 (1.08–1.59)	1.07 (0.88–1.30)	1.00 (ref.)	0.0001
Yes	1.37 (1.05–1.78)	1.06 (0.81–1.38)	1.09 (0.83–1.44)	0.88 (0.66–1.17)	0.023	1.81 (1.45–2.26)	1.49 (1.19–1.87)	1.08 (0.85–1.37)	0.91 (0.71–1.16)	<0.0001
Type 2 diabetes										
No	1.43 (1.15–1.78)	1.13 (0.91–1.40)	1.12 (0.91–1.39)	1.00 (ref.)	0.009	1.62 (1.36–1.93)	1.39 (1.17–1.64)	1.11 (0.94–1.32)	1.00 (ref.)	<0.0001
Yes	1.84 (1.37–2.47)	1.38 (1.00–1.89)	1.33 (0.95–1.87)	1.35 (0.96–1.90)	0.120	1.97 (1.51–2.58)	2.06 (1.54–2.75)	1.37 (0.99–1.91)	1.26 (0.90–1.77)	0.415
Dyslipidemia										
No	1.45 (1.13–1.87)	1.12 (0.87–1.43)	1.04 (0.80–1.34)	1.00 (ref.)	0.054	1.58 (1.29–1.92)	1.54 (1.28–1.87)	1.06 (0.87–1.28)	1.00 (ref.)	<0.0001
Yes	1.35 (1.04–1.75)	1.07 (0.82–1.40)	1.13 (0.87–1.48)	0.98 (0.74–1.29)	0.012	1.74 (1.39–2.16)	1.28 (1.02–1.60)	1.28 (1.01–1.61)	1.05 (0.83–1.33)	0.015

CI, confidence interval; PUFA, Polyunsaturated fatty acid. Prevalence was conducted by Cochran–Mantel–Haenszel analysis with adjustment for age. Odd ratios and 95% CIs were conducted by general linear model analysis with adjustment for age, body mass index, menarche (women only), education level, income status, alcohol intake, smoking, exercise, total energy intake, hypertension, diabetes, and dyslipidemia.

## Data Availability

Data are available from the Korea National Health and Nutrition Examination Survey (KNHANES VII and VIII), conducted by the Korea Disease Control and Prevention Agency (KDCA), and are freely available from KDCA (https://knhanes.cdc.go.kr, accessed on 1 April 2022).
